# Evidence of Melanoma in Wild Marine Fish Populations

**DOI:** 10.1371/journal.pone.0041989

**Published:** 2012-08-01

**Authors:** Michael Sweet, Nigel Kirkham, Mark Bendall, Leanne Currey, John Bythell, Michelle Heupel

**Affiliations:** 1 Coral Health and Disease Laboratory, School of Biology, Newcastle Institute for Research on Sustainability, Newcastle University, Newcastle upon Tyne, United Kingdom; 2 Cellular Pathology, Royal Victoria Infirmary, Newcastle upon Tyne, United Kingdom; 3 AIMS@JCU, Australian Institute of Marine Science, School of Earth and Environmental Sciences, Fishing and Fisheries Research Centre, James Cook University, Townsville, Australia; 4 Research Office, University of the South Pacific, Suva, Fiji; 5 Australian Institute of Marine Science, Townsville, Australia; 6 Fishing and Fisheries Research Centre, School of Earth and Environmental Sciences, James Cook University, Townsville, Australia; Institut Pasteur, France

## Abstract

The increase in reports of novel diseases in a wide range of ecosystems, both terrestrial and marine, has been linked to many factors including exposure to novel pathogens and changes in the global climate. Prevalence of skin cancer in particular has been found to be increasing in humans, but has not been reported in wild fish before. Here we report extensive melanosis and melanoma (skin cancer) in wild populations of an iconic, commercially-important marine fish, the coral trout *Plectropomus leopardus.* The syndrome reported here has strong similarities to previous studies associated with UV induced melanomas in the well-established laboratory fish model *Xiphophorus*. Relatively high prevalence rates of this syndrome (15%) were recorded at two offshore sites in the Great Barrier Reef Marine Park (GBRMP). In the absence of microbial pathogens and given the strong similarities to the UV-induced melanomas, we conclude that the likely cause was environmental exposure to UV radiation. Further studies are needed to establish the large scale distribution of the syndrome and confirm that the lesions reported here are the same as the melanoma in *Xiphophorus*, by assessing mutation of the EGFR gene, Xmrk. Furthermore, research on the potential links of this syndrome to increases in UV radiation from stratospheric ozone depletion needs to be completed.

## Introduction

Prevalence and occurrence of novel diseases are reported to be increasing in many organisms worldwide. Understanding the etiology of these diseases, the host organisms they affect and potential causes and consequences are a vital first step in the development of control and management strategies. Many diseases are caused by microbial pathogens, and fish diseases in particular have been shown to be caused by a diversity of such pathogens including bacteria, parasitic copepods, viruses and fungi [1], [2], [3]. Historically, diseases in fish have been recorded more commonly in species of commercial value, usually farmed fish. This may be due to the higher than normal stocking densities which in turn can lead to higher levels of infections and/or the ease of sampling large numbers and continuous monitoring capabilities. Furthermore, there is also significant economic benefit to identifying pathogens of these commercially reared fish with the aim of ultimately curing them. In aquaculture systems, diseases cause a significant economic loss, with bacteria, viruses and fungi being the dominant pathogens involved [1], [3]. In contrast, diseases of wild fish have received considerably less attention and their economic impact on commercial and recreational fisheries is unknown. In addition to microbial diseases common in fish, other diseases such as carcinomas have been extensively studied in the laboratory using fish model systems, including the *Xiphophorus* (swordtail) [4], [5] and, more recently, the *Danio* (zebrafish) models [5], [6]. To date, however, there are no reports of cancers occurring in wild fish populations. This study aimed to describe a previously unknown disease lesion, which was observed affecting large numbers of a commercially important reef fish, the coral trout *Plectropomus leopardus*.

## Methods

### Sampling

Individual coral trout, *Plectropomus leopardus*, were line caught with barbless 8/0 hooks using pilchard bait, following methods employed by commercial fishers. Four fishing trips were completed between Aug 2010 and Feb 2012 off the east coast of Australia, at Heron Island (23.4°S, 151.9°E) and One Tree Island (23.5°S, 152.0°E). In total 136 fish were sampled and photographed, 20 of which showed signs of skin abnormalities. From healthy individuals and those with the syndrome, two sets of samples were taken; one for microbial analysis and the other for histological examination. Additional affected individuals were observed during snorkel and dive activities, but only those individuals captured via fishing were included in this analysis.

**Figure 1:**
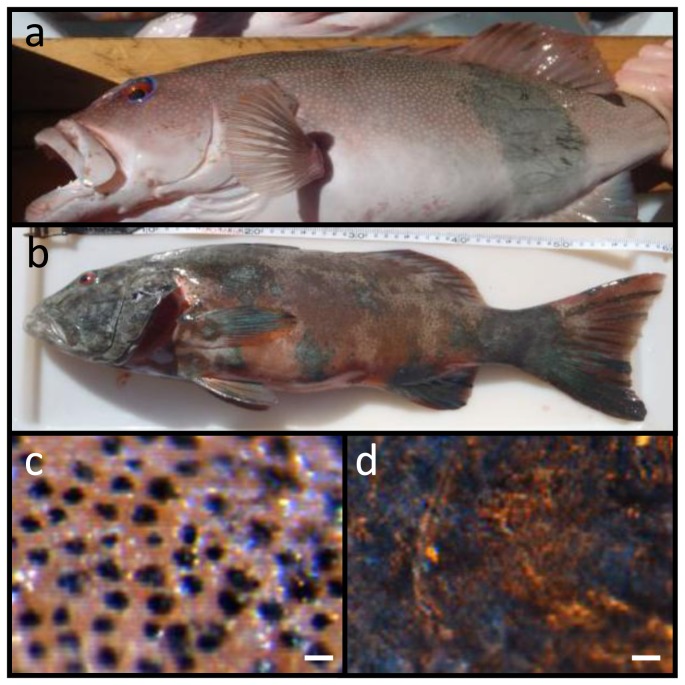
Lesions were present on approximately 15% of the sampled population of *Plectropomus leopardus*; a) affected individual showing <10% coverage of body surface; b) *P. leopardus* with almost complete coverage >90%; c) healthy tissue under light microscope and d) the lesion. Scale bars = 20 µm.

To test for differences in bacterial, fungal and ciliate molecular diversity between healthy and lesion samples, we analyzed tissue sections collected from individuals captured in August 2011. Three replicate tissue sections (∼10l×3 w×3 d cm), separated by ∼5 cm were cut using a sterile scalpel blade from n = 5 non-diseased (ND) fish, n = 5 diseased (D) fish (Fig. 1a) at the lesion interface and n = 5 apparently healthy (AH) tissues adjacent to the lesion on a disease fish. Samples were placed directly into 100% EtOH and stored at −20°C until extraction and further analysis. A further set of samples, aimed at sampling the surface associated microbes, utilised sterile swabs. The surface of the fish scales were swabbed and these were placed directly into sterile micro-centrifuge tubes with 100% EtOH, stored at −20°C until extraction and further analysis. Further samples, aimed at sampling the surface associated microbes used sterile swabs. These were placed in sterile micro-centrifuge tubes and stored in 100% EtOH at −20°C until extraction. Samples for histology were collected as for microbial analysis (see above), with the same sample number of samples however, they were preserved in 5% paraformaldehyde made up with Phosphate Buffer Saline (PBS). Samples were fixed for 24 hr, dehydrated in a dilution series of EtOH from 50 to 100% and stored at 4°C until embedding and sectioning. DNA was extracted using the QIAGEN DNeasy Blood and tissue extraction kit.

### Fungal PCR amplification and denaturing gradient gel electrophoresis (DGGE) of tissue samples/swabs

For DGGE analysis a portion of the fungal ITS rRNA gene was amplified using universal fungal primers; a nested PCR approach was utilised to yield the most complete diversity [7]. 1^st^ round; fungal primers ITS1F (5′CTTGGTCATTTAGAGGAAGTAA-3′) and ITS4 (5′-TCCTCCGCTTATTGATATGC-3′) [8] were used following the protocol described by [9] (94°C for 5 min; 35 cycles at; 94°C for 30 sec, 55°C for 30 sec, 72°C for 30 sec then elongation at 72°C for 5 min). 20 µl PCR reactions were routinely used (final PCR buffer contained: 1 mM MgCl_2_, and 1 U Taq DNA polymerase (QBiogene); 100 µM dNTPs; 0.2 µM of each of the forward and reverse primers; and 0.4% BSA, with 20 ng of template DNA). A 1∶100 dilution of the PCR product was then used in a further PCR with the primers ITS3 (5′-GCATCGATGAAGAACGCAGC-3′) and ITS4-GC (5′-CGCCCGCCGCGCCCCGCGCCCGGCCCGC CGCCCCCGCCCC-TCCTCCGCTTATTGATATGC -3′) [10]. All reactions were performed using a Hybaid PCR Express thermal cycler. PCR products were verified by agarose gel electrophoresis [1.6% (w/v) agarose] with ethidium bromide staining and visualized using a UV transilluminator. DGGE was performed using the D-Code universal mutation detection system (Bio-Rad). PCR products were resolved on 10% (w/v) polyacrylamide gels that contained a 30–60% formamide (denaturant) gradient for 13 h at 60°C and a constant voltage of 50 V. Gels were stained with SYBER gold as described by [11]. Bands of interest (those which explained the greatest differences/similarities between samples) were excised from DGGE gels, left overnight in Sigma molecular grade water, vacuum centrifuged, re-amplified with the specific primers, labelled using Big Dye (Applied biosystems) transformation sequence kit and sent to Genevision (Newcastle University, UK) for sequencing. Fungal operational taxonomic units (OTUs) were defined from DGGE band-matching analysis using BioNumerics 3.5 (Applied Maths BVBA).

### Bacterial PCR amplification and denaturing gradient gel electrophoresis (DGGE) of tissue samples/swabs

Extraction was the same as above. For DGGE analysis a portion of the bacterial 16S rRNA gene was amplified using universal eubacterial primers [12]; (357F-GC) (5′-CCTACGGGAGGCAGCAG-3′) and (518R) (5′- CGCCCGCCGCGCGCGGCGGGCGGGGCGGGGGCAGCACGGGGGG-ATTACCGCGGCTGCTGG-3′). PCR reaction mixtures and program were as described by [11]. PCR products were resolved on 10% (w/v) polyacrylamide gels that contained a 30–60% formamide (denaturant) gradient for 13 h at 60°C and a constant voltage of 50 V. Gels were stained as above and bands of interest were excised from DGGE gels, labelled and sent to Genevision (Newcastle University, UK) for sequencing. Bacterial OTUs were defined from DGGE band-matching analysis using Bionumerics 3.5 (Applied Maths BVBA).

### Ciliate PCR amplification and denaturing gradient gel electrophoresis (DGGE) of tissue samples/swabs

Ciliates 18S rRNA genes were amplified with an un-nested PCR approach. PCR mixture was as above with the forward primer CilF (5′-TGGTAGTGTATTGGACWACCA-3′) with a 36-bp GC clamp [13] attached to the 5′ end and CilDGGE-r (5′TGAAAACATCCTTGGCAACTG-3′). Initial denaturation was at 94°C for 5 min, followed by 26 cycles of 94°C for 1 min, 52°C for 1 min, and 72°C for 1 min and a final elongation step of 10 min at 72°C to reduce double bands in the DGGE patterns. The DGGE was carried out using a D-code system (Bio-Rad) with 0.75 mm thick 6% polyacrylamide gels in 1× TAE buffer. Electrophoresis was carried out for 16 h at 60°C and 50 V in a linear 32 to 42% denaturant (formamide) gradient. Gels were stained as above.

### Statistic analysis of microbial communities

In order to assess variation in microbial assemblages (bacterial, fungal and ciliate), matrices consisting of OTU's and samples were generated using both presence/absence and band intensity data, using marker lanes for between-gel comparisons. Changes in microbial assemblages were evaluated with a one-way analysis of similarity (Primer, ANOSIM) and multi dimensional scaling (MDS), based on Bray-Curtis similarities, which was performed on all samples sets, healthy, apparently healthy and diseased.

### Histology

Samples were collected as for microbial analysis; however tissue samples were preserved with 5% paraformaldehyde for 24 hrs then stored in 100% EtOH until resin embedding in LR white ^(r)^. For each tissue type, the location of bacteria was recorded using fluorescent *in situ* hybridisation (FISH) and the general fluorescent stain, 4′6-diamidino-2-phenylindole (DAPI). For FISH, samples were stained and sectioned following the protocols in [14], with the addition of an equimolar mix (EUBMIX). Oligonucleotide probes were purchased from Interactiva (http://www.interactiva.de) with an aminolink C6/MMT at the 5′ end. Four probes were used: the ‘universal’ eubacterial probes EUB338 (5′-GCT GCC TCC CGT AGG AGT-3′), EUB338-II (5′-GCA GCC ACC CGT AGG TGT-3′), EUB338-III (5′-GCT GCC ACC CGT AGG TGT-3′) and the ‘non-sense probe’ NONEUB (5′-ACT CCT ACG GGA GGC AGC-3′), which has the complementary sequence to EUB338, used to determine non-specific binding of EUB338. The three eubacterial probes were used in an equimolar mix (EUBMIX) and the NONEUB probe was used singly. DAPI staining followed the protocol by [11], whereby each section was stained with 100 µl of 4% PBS buffered paraformaldehyde solution containing 4′6-diamidino-2-phenylindole (final concentration 5 µg ml^−1^) for 10 minutes, rinsed with filtered 1× PBS pH 7.4. All sections were viewed under epiflourescence microscopy with an FITC-specific filter block (Nikon UK Ltd, Surrey, UK) and images recorded using an integrating camera (Model JVC KY-SSSB: Foster Findlay and Associates, Newcastle upon Tyne, UK). Samples of pure cultured *E.coli* were run alongside each section and for each staining protocol as a positive stain.

Further histological samples were stained with the melanin specific stain, Fontana-Mason, melanin granules reduce silver nitrate to metallic silver, which results in a histochemical reaction that participates black material wherever melanin is located [15].

Samples for Scanning Electron Microscopy (SEM) were dehydrated using EtOH and PBS; 25% EtOH, 50% EtOH, 75% EtOH (30 mins each), then a further (2×1 hr) in 100% EtOH, with final dehydration using carbon dioxide in a Baltec Critical Point Dryer. Specimens were then mounted on an aluminium stub with Achesons Silver Dag (dried overnight) and coated with gold (standard 15 nm) using a Polaron SEM Coating Unit. Specimens were examined using a Stereoscan 240 Scanning Electron Microscope, and digital images collected by Orion6.60.6 software.

Samples for Transmission Electron Microscopy (TEM) were dehydrated using 25% acetone, 50% acetone, 75% acetone, (30 min each) and 100% acetone (2×1 h). Then impregnated with 25% LR White resin in acetone, 50% resin/acetone, 75% resin/acetone (1 h each), then 100% resin for minimum of 3 changes over 24 h, with final embedding in 100% resin at 60°C for 24 hrs. Survey sections of 1 µ were cut and stained with 1% Toluidine Blue in 1% Borax. Ultrathin sections (80 nm approx) were then cut using a diamond knife on a RMC MT-XL ultramicrotome. These were then stretched with chloroform to eliminate compression and mounted on Pioloform filmed copper grids. Staining was with 2% aqueous Uranyl Acetate and Lead Citrate (Leica). The grids were then examined using a Philips CM 100 Compustage (FEI) Transmission Electron Microscope and digital images were collected using an AMT CCD camera (Deben) at the Electron Microscopy Research Services Laboratory, Newcastle University.

## Results and Discussion

Approximately 15% of samples from a population of *Plectropomus leopardus* line caught at two locations in the southern Great Barrier Reef Marine Park - Heron Island and One Tree Island - showed evidence of a dark growth lesion (Fig. 1a,b), similar in appearance to those reported from laboratory induced melanomas seen in the fish *Xiphophorus* [16]. Prevalence of skin lesions was not significantly different (Chi Square = 0.063, df = 1, p = 0.803) between reef platforms with 14.1% of individuals at Heron Island and 15.7% of individuals at One Tree Island affected. In this study, the fish displaying these skin lesions struck fishing hooks as strongly as healthy individuals, appeared to have good muscle tone and were assessed by external examination as healthy aside from the skin discolouration. Coverage of the lesion on individual fish varied from <10% of body surface (Fig. 1a) to almost complete coverage (Fig. 1b). Although the size range of individuals sampled was limited (344–639 mm fork length), there was no relationship between percent cover and fish size (r^2^ = 0.02). Small individuals (468 mm) could show up to 98% lesion cover and larger individuals (639 mm) showed as little as 30% cover, indicating that prevalence can occur at varying sizes and ages. Lesions affected the surface of the fish caught, with a change from the characteristic blue-spotted patterning (Fig. 1c) in healthy individuals to raised lesions which were darker black/brown in coloration (Fig. 1d). Location of the lesions on the body varied between individuals.

### Associated microbial (rRNA gene) communities

**Figure 2:**
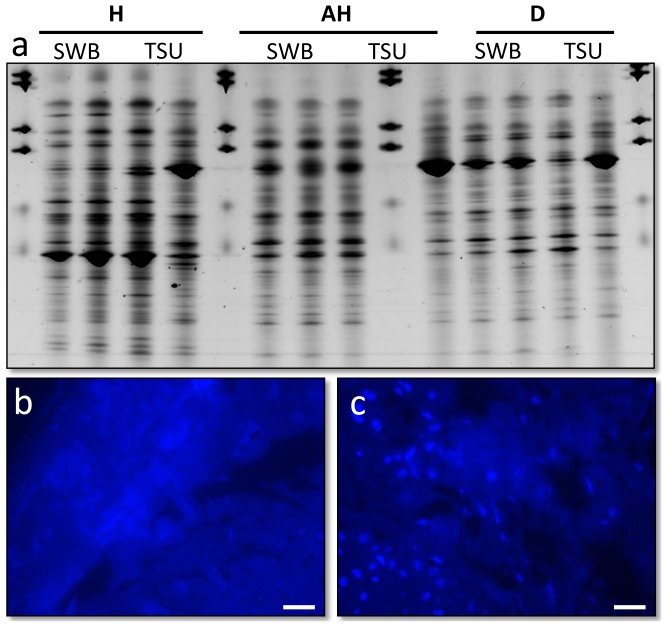
Microbial analysis of *Plectropomus leopardus* samples; a) Bacterial 16S rRNA gene fingerprints (represented on Denaturing Gradient Gel Electrophoresis) of fish mucus (SWB) and tissue samples (TSU), standardised for gel-to-gel comparison using BioNumerics; b) resin embed histological section of a healthy fish, stained with the general DNA stain 4′6-diamidino-2-phenylindole (DAPI); c) histological section of the lesion on a diseased fish stained with DAPI, both showing no bacteria within the dermis suggesting the bacteria present in (a) are localised within the mucus layer not within the tissues. Scale bars = 10 µm.

**Figure 3:**
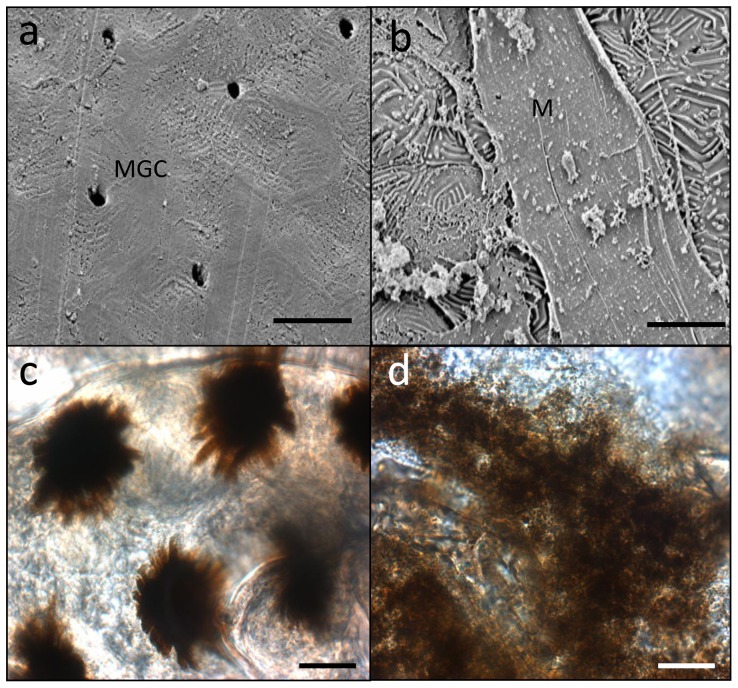
Microscopic images of *Plectropomus leopardus* tissues; a) Scanning Electron Micrograph (SEM) of the healthy tissue; b) SEM of the lesion. MGC = mucus goblet cells, M = mucus. c) Light microscope image of a healthy scale and d) light microscope image of a diseased scale, showing disorganisation of natural melanin patterns seen in (c). Scales bars = 10 µm.

**Figure 4:**
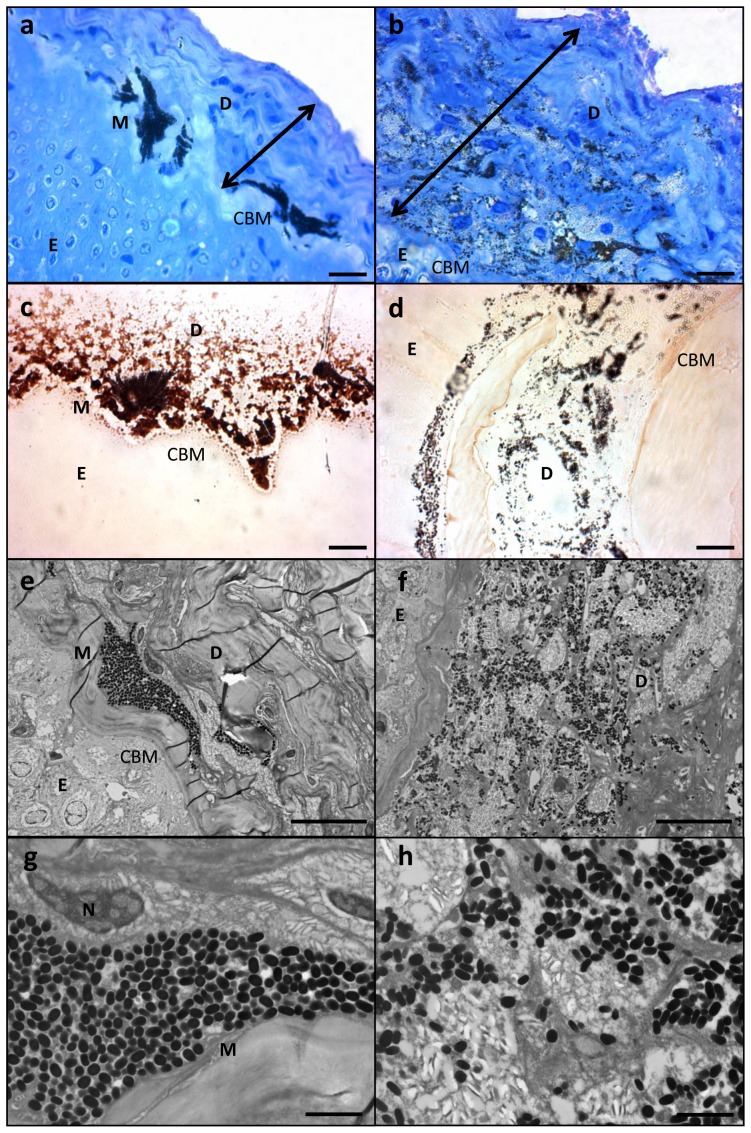
Histological section of LR white resin embedded samples of healthy and diseased *Plectropomus leopardus*; a) Healthy section stained with toluidine blue; b) lesion stained with toluidine blue; c) healthy section stained with melanin specific stain Masson-Fontana; d) lesion stained with Masson-Fontana; e) Transmission Electron Micorgraph (TEM) of healthy section; f) TEM of lesion; g) higher magnification of TEM in (e); h) higher magnification of TEM in (f). Scale bars for (a–f) = 10 µm; scale bars for (g) and (h) = 2 µm. D = dermis (cologne of stroma), E = epithelium, M = melanosome, N = cell nucleus, CBM = caliginous basal membrane. Double headed arrows shows thickening of the integument, characteristic of laboratory induced-melanomas in the *Xiphophorus* model.

Analysis of microbial communities associated with healthy (non-diseased) and diseased fish, which would highlight potential pathogenic agents (those present in lesions and absent in healthy samples [17], [18]), was conducted using culture-independent (rRNA gene) molecular screening techniques. Swabs of the mucus and tissue samples from healthy fish, apparently healthy tissues on affected fish, and the lesion itself were sampled. Microbial (rRNA gene) diversity assessed using bacterial-, fungal- and ciliate-specific PCR primers showed no significant difference (p>0.45) between the sample types. No known microbial pathogen sequences were found in lesion samples that were absent or in lower numbers within healthy and/or apparently healthy samples (Fig. 2a). The technique utilised in this study has routinely been used successfully in other studies to highlight potential microbial pathogens [19], [20], [21]. Furthermore, no significant differences (p = 0.12) was found between tissue sections and non-invasive surface mucus swabs, suggesting that the microbial communities detected were mainly present on the surface of the fish and not in the dermis or muscle tissues, where the lesion recorded. Histological sections visualised with either Fluorescence *In Situ* Hybridisation (FISH) using eubacterial probes [14] or the general nucleic acid stain DAPI (Fig. 2b,c), showed no microbial populations within the dermis, which supports the conclusion that the microbes detected using culture-independent screening were associated with the surface mucus layer of the fish. No evidence of these or other microbes such as virus like particles (VLPs) were detected using either Scanning Electron Microscopy (SEM) for surface microbes (Fig. 3a,b), or Transmission Electron Microscopy (TEM) (Fig. 4e, f, g, h) for those within the tissues. Processing for SEM and TEM would have removed the surface mucus layer; again supporting the conclusion that few, if any, microbes were present within the dermis at the sites of pathogenesis.

### Histopathological analyses

**Figure 5:**
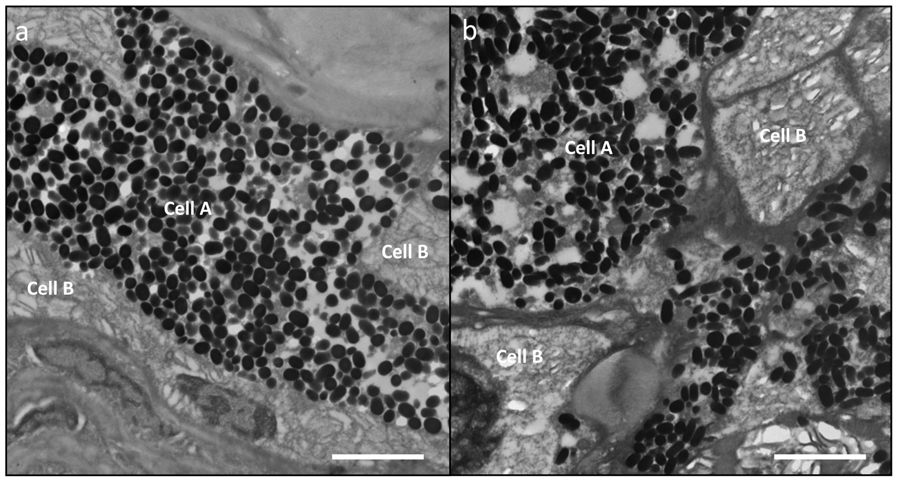
Transmission Electron Micrographs of different samples of *P. leopardus* exhibiting; (a) healthy tissue showing the two cell types (A & B) associated in the dermis along the collagenous basal membrane (CBM). Cell A shows localisation of melanosomes and Cell B shows absence of melanosomes in the same area. (b) Lesion showing disorganisation of pleomorphic cells (A & B) with an increase in number and spread of melanosomes. This lesion is an example of a *P. leopardus* suffering from stage II melanoma, where the melanosomes are restricted to the dermis. Scale bars = 10 µm.

Melanin-containing cells (melanosomes) were found to be in higher density, more widespread and with a deeper distribution within the lesions than compared to healthy tissue sections (Fig. 1c, d and Fig. 3c, d). In normal fish skin these cells are restricted to the immediate subepithelial dermis and are responsible for the pigment patterns in the integument [22]. Melanosomes are normally found to be well organised and clustered in tight groups throughout the dermis beneath the epidermal basement membrane (Fig. 4a, c, e, g). Sections from apparently healthy areas of skin from affected fish showed this normal pattern, whilst samples from lesional plaques, which often occurred in areas that are not normally pigmented, showed a tumourous appearance of disorganised pleomorphic cells containing melanosomes (Fig. 4b, d, f, h). Melanosomes in the lesions contained more pigment and were thought to be mature, older cells [5]. The number of melanosomes, and hence pigmentation, in the cells varied from completely absent (Cell B) to cells with plentiful melanosomes (Cell A). Melanin-specific Masson-Fontana-stained sections (Fig. 4c, d) were used to visualise these melanin-producing pigmented cells. A thickening of the integument (Fig. 4a,b double headed arrows) and extensive melanosis (development of melanotic overgrowths, which in turn is a consequence of extreme pigment cell proliferation), can clearly be seen in the cases of all lesion samples in this study and are characteristic of laboratory induced-melanomas in the *Xiphophorus* model [16]. Usually there is little distinction between premalignant melanosis and melanomas, whereby in the former the number of pigmented cells (melanophores) is increased but restricted to the dermis (as was the case for most of the lesions in this study), and in the latter the melanophores invade the underlying tissues. However, 5 prominent types of melanomas have previously been distinguished [16], one of which Melanophorous-Macromelanophorous Polymorphic Melanoma (MMPM) is known to be heterogeneous, with heavily and lightly pigmented areas, as observed here. Lesions contained different cell types, including melanocytes, epitheliod-like cells, melanophores and macromelanophore cells (Fig. 4f,h and Fig. 5b), consistent with MMPM. Interestingly, the majority of *P. leopardus* examined, exhibited lower density and coverage of skin lesions (Fig. 1a). However, this may be due to the sampling regime utilised in this paper. This was further reflective histopathologically, with stage I or stage II melanomas as described by [23], where the macromelanophores were restricted to the dermis, the meninges, the peritoneum, and the perivascular connective tissue of the blood vessels (Fig. 5b). No fish analysed in this study showed a more advanced stage of melanoma development, stage III, IV and V, where the macromelanophores penetrate the *stratum compactum* of the dermis and invade the underlying muscles. Fishes exhibiting this more advanced stage may show behavioural differences in the wild and may therefore have not been caught using the techniques utilised in this study. Further work to follow disease progression on captive held individuals would highlight the spread of the lesion, show the different stages of cancer, and show whether this type of melanoma is benign or malignant.

Given the strong histopathological similarities between the lesions described here in *P. leopardus* and the UV radiation-induced melanomas in the laboratory model *Xiphophorus* [5], [22], along with a lack of any evidence for a pathogenic cause, we conclude that this represents the first case of melanoma in a wild fish population. As the sampled fish were collected offshore in a marine protected area with no reports of pollution, the likelihood of potential carcinogenic pollutants being the causal factor is low, at least in this reported case. UV radiation, in comparison, is known to be a causal factor in skin damage in many animals and therefore is a likely driving factor of prevalence of melanoma in *P. leopardus*. There is a significant correlation between average solar radiation (i.e. latitude) and melanoma mortality in humans [5], [24]. UV-B (λ = 280–320 nm) appears to be the most damaging radiation [25] and has previously been shown to increase in intensity as stratospheric ozone levels have decreased [26]. UV radiation in aquatic systems has previously been reported to have detrimental effects on marine and freshwater organisms, with UV penetrating as deep as 60 m in the sea [27], [28], [29]. Therefore *P. leopardus* inhabiting the clear waters of the Great Barrier Reef would be exposed to UV radiation over a wide depth range. Individuals in this study were all captured in less than 20 m depth, well within the UV-B exposure range of 30 m [27]. Interestingly, juvenile hammerhead sharks have been shown to have the ability to ‘sun tan’ [30], whereby integumental pigments such as melanin increased in direct response to increases in solar radiation. The juvenile shark's skin responded similarly to that observed in humans and other vertebrates in response to direct sunlight, turning from brown to black. Although a similar melanin response was seen in this study (i.e. increased melanin concentration), the sharks in this previous study showed no visible lesions or growths and were therefore not shown to contract melanomas or dermal carcinomas.

With regard to the *Xiphophorus* induced melanoma model, it had long been assumed that only hybrid crosses of *Xiphophorus* (those bred in captivity), could be induced to contract melanomas or experience extensive melanosis. The wild (parental) types of these species in comparison, were non-susceptible to neoplasia, even after exposure to high doses of physical and chemical carcinogens [4], [31]. However, in addition to this study illustrating melanosis/melanoma induction in wild type *Plectropomus*, one further study on *Xiphophorus* also showed non-hybrid melanoma formation in a wild caught fish, however this was accredited to a build up of androgen metabolites within the holding tank [22]. Hybrid strains of *Xiphophorus* have been noted to have differing susceptibility to carcinogens suggesting a genetic basis for susceptibility to melanoma formation [32]. Furthermore, it has been shown that melanoma in *Xiphophorus* is caused by a mutated EGFR gene, *Xmrk*, with constitutive expression of growth factors. When *Xmrk*, is transplanted into another fish *Oryzias latipes*, they subsequently contract melanomas themselves [33], [34]. Therefore, this suggests an underlying genetic predisposition to the disease that is expressed with the loss of tumour suppressor genes caused by the onset of hybridisation. The occurrence of melanoma in a wild population, particularly, at the levels observed in this study is unusual. The relatively high (15%) prevalence of this syndrome within the sampled *P. leopardus* population may be indicative of a similar genetic defect as that experienced during hybridisation in the laboratory, or alternatively it may be due to potential inbreeding in this portion of the *P. leopardus* population resulting in recessive susceptibility genes becoming homozygous. In the latter instance, inbreeding may be potentially proliferated in the local area due to recruitment of genetically related individuals to the same reef system [35]. However, hybridisation has frequently been shown to occur in wild populations of many fish species [36], [37], including populations of *P. leopardus* which have been shown to hybridise with other *Plectropomus* species, such as the Bar-cheeked coral trout, *P. maculatus* [38]. Frisch and van Herwerden (2006) concluded that despite behavioural barriers to reproduction (such as assortative mating), there was considerable opportunity for hybridisation between different species of coral trout. Indeed, the same macroscopic signs of this disease have been noticed on *P. maculates* and one further species, the blue spotted coral trout, *P. laevis*, suggesting hybridisation between these species may be the most likely cause of predisposition of *Plectropomus* to melanomas. Current information suggests this syndrome is present throughout the Great Barrier Reef (MRH unpublished data), but prevalence appears to be highest in the southern Great Barrier Reef. This high prevalence recorded in this study further supports the presence of a genetic component to this syndrome, yet detailed, broader sampling is required to confirm the extent of prevalence in other Great Barrier Reef regions.

Coral trout, *P. leopardus*, is an iconic and highly valued species and the Great Barrier Reef is one of the world's most pristine and carefully managed reef habitats. Successful management of these resources is a crucial and challenging task [39]. The implications of extensive melanosis/melanoma in wild coral trout will depend on the prevalence of the syndrome outside the study region, the causal factors and the proportion which develop into fatal melanomas. However, this syndrome will no doubt have implications for the management of fish populations and the GBR marine park. Beyond health implications for individual fish, this syndrome may have implications for the population as a whole and the commercial and recreational fisheries that exploit this species. In *Xiphophorus*, fish with tumours usually survive around 6 months, compared to an average of 4 years in healthy fish, but any change in their environment, such as a drop in temperature can rapidly lead to death [5]. It is unclear whether future changes in the ocean environment or climate will similarly exacerbate the effect of melanomas in wild *P. leopardus* populations, but clearly further research is urgently needed to understand the distribution, prevalence, ecological and fisheries significance of this syndrome. In particular, further studies should focus on UV exposure as a risk factor and confirm whether there is a genetic effect to susceptibility of the syndrome. Utilising molecular markers used to study melanomas in humans and laboratory fish models e.g. those that target the B-Raf protein [40], the EGFR gene, *Xmrk*, or other mitochondrial DNA status markers [41] would highlight this genetic aspect.
